# Mini gastric bypass for the management of gastrobronchial fistula: A case report

**DOI:** 10.1016/j.ijscr.2019.11.064

**Published:** 2019-12-09

**Authors:** Abdulhamid Alharbi, Mohammed Alnaami, Abdulrahman Alsayyari, Mana Almuhaideb

**Affiliations:** a Department of Surgery, King Khalid University Hospital, P.O. Box 7805 #37, Riyadh, 11472, Saudi Arabia; b College of Medicine, King Saud University, P.O. Box 7805 #37, Riyadh, 11472, Saudi Arabia

**Keywords:** Case report, Gastrobronchial fistula, Mini gastric bypass, Laparoscopic sleeve gastrectomy, Obesity, Fistulo-Jejunostomy

## Abstract

**Introduction:**

With the high rates of obesity worldwide, laparoscopic sleeve gastrectomy (LSG) has become a very popular procedure. Due to its simple technique, rare complications might be overseen. Gastric leaks and fistula are fairly uncommon complications. In comparison to other types of fistulas, gastrobronchial fistulas are rarer with serious complications. Definitive management is yet to be determined. We intend to explore the literature on the management approach of such patients.

**Presentation of case:**

A 46-year-old male, presented with on/off abdominal pain, productive cough, and vomiting. The patient had left sided rhonchi on examination. In addition to a history of laparoscopic sleeve gastrectomy (LSG) 4 years ago. Imaging confirmed the presence of a gastrobronchial fistula. Conservative and endoscopic treatment failed. Consecutively, surgery was indicated. A laparoscopic mini gastric bypass with refashioning of gastric fistula edges and closure with graham patch was done.

**Conclusion:**

Given the increasing number of such surgeries performed the recognition of acute and chronic complications, and their optimal management is of great importance. Although performing a Roux-en-Y fistulojejunostomy was recommended in the literature, conservative and endoscopic treatment should be considered before.

## Highlights

• Gastrobronchial fistula is uncommon with an ambiguous presentation.
• It should be suspected among patients who underwent bariatric surgery.
• Optimal management is yet to be determined.

## 1 Introduction

Laparoscopic sleeve gastrectomy (LSG) has become a common bariatric procedure, and its popularity is growing by the day. The simplicity of the procedure camouflages a number of serious, sometimes fatal, complications. Gastric leaks are a fairly uncommon complication with a rate that can reach up to 8% [1]. Perpetuation of the leak might lead to the development of a fistula [2]. Developing a fistula is even rarer, with rates ranging from 2% to 4% [3]. Causes of leaks can be classified as mechanical, technical and ischemic. The most important clinical signs and symptoms in patients with gastric leaks are fever and tachycardia [4]. The management options include endoscopic interventions, surgical intervention, or conservative management. In this paper, we present a case of a 46 years old male, who underwent LSG which was complicated later with a gastrobronchial fistula. The work has been reported in line with the SCARE criteria [5].

## 2 Case presentation

A 46 years old male was seen in the outpatient clinic with the complains of on/off abdominal pain, productive cough with yellow sputum, and vomiting occasionally mixed with blood. There was no fever or night sweat. In addition to, an unremarkable systematic review.

On physical examination, the patient was in mild pain. With rhonchi heard at the left lower chest. The abdomen was soft and lax with mild tenderness in the left upper quadrant.

Past surgical history was significant for laparoscopic sleeve gastrectomy 24 months ago where the patient had a BMI of 44.68 kg/m^2^ and was discharged 2 days after with no complication. Labs were ordered and showed the following: Red blood cell count of 4.6 10^12^/L, white blood cell count of 6.1 10^9^/L, a hemoglobin level of 115 g/L, and platelet level of 227 10^9^/L.

**Fig. 1:**
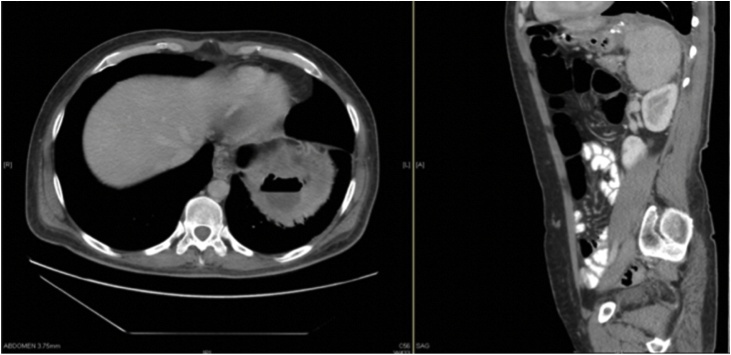
Axial and Sagittal CT view showing a left subphrenic collection showing an air-fluid level associated with mild thickening of the diaphragmatic crus and linear atelectatic changes of the adjacent lung.

Earlier that year, the patient presented to the emergency department complaining of generalized abdominal pain and was found to have a splenoportovenous thrombosis. Subsequently, the patient was referred and started following up with the hematology clinic. A follow up CT showed an incidental finding of a left subphrenic focal pouch measuring 3.5 cm with air fluid level. Since the patient was asymptomatic at that time, no intervention was proposed. A later CT was done (Fig. 1) and showed an increase in size in comparison to the previous study, with air-fluid level associated with stable left diaphragmatic and pleural thickening, with subsequent linear adjacent basal lung atelectasis.

**Fig. 2:**
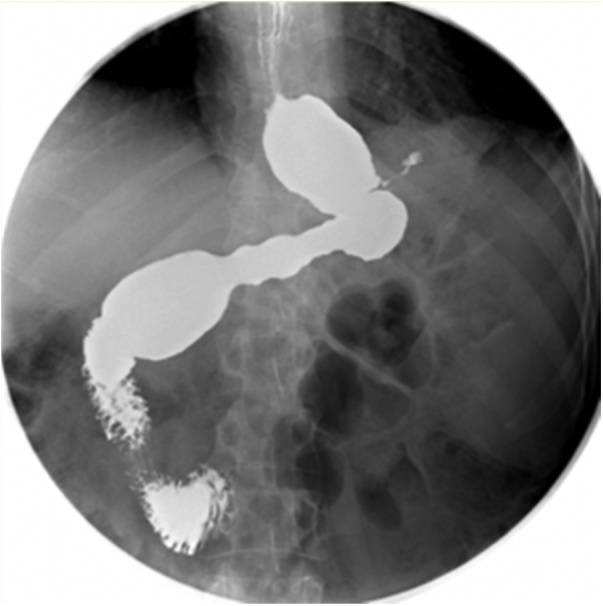
X-ray fluoroscopy barium meal revealing linear contrast leak arising from the left posterior lateral aspect of the proximal gastric pouch, extending to the left sub-diaphragmatic region. No communication with the pleural space at that time.

To further define the extent of the leak, an X-ray fluoroscopy barium meal (Fig. 2) was done and confirmed a leak from the proximal gastric pouch with no communication to the pleural space. Percutaneous drainage was placed by interventional radiology (IR) which showed bloody flanks with thick pus. The patient was discharged with a follow up in the clinic where he came later complaining of unresolving cough with undocumented fever.

**Fig. 3:**
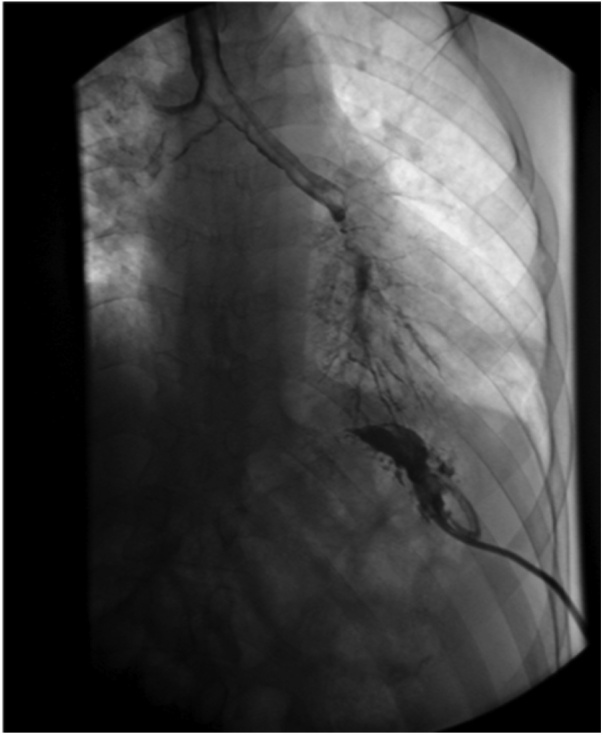
X-ray fluoro-fistulogram showing contrast from the left upper quadrant collection to be freely communicating with the left lower lobe bronchioles reaching into the left main bronchus and trachea.

An X-ray fluoro-fistulogram (Fig. 3) showed the contrast from the left upper quadrant collection appears to be freely communicating with the left lower lobe bronchioles reaching into the left main bronchus and trachea. And the diagnosis of a gastrobronchial fistula was established.

Endoscopic management was initially trialed. Nevertheless, stent placement was aborted during endoscopy due to the presence of esophageal varices. Instead, an over the scope clip (OTSC) was placed with no complication. Thereafter, the clip was unable to close the whole fistula. And surgery was indicated.

**Fig. 4:**
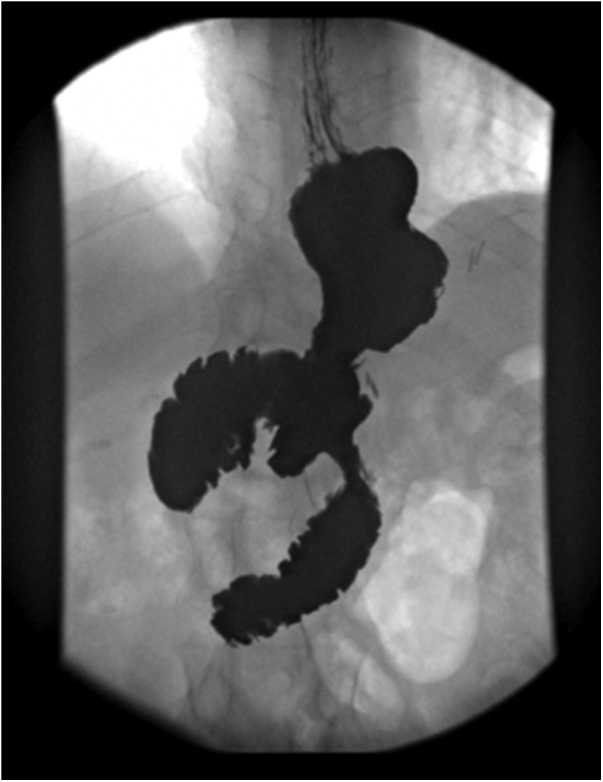
Gastrographin study 6 months after the procedure showing the absence of any leak.

Under general anesthesia, the patient was placed supine with legs apart. Surgery was performed by an attending and professor of upper gastrointestinal surgery with the assistance of an upper gastrointestinal surgery fellow. Laparoscopic approach was carried out through 5 ports. A 12 mm camera port placed supraumbilical, two 12 mm ports placed in the right and left lateral, a 5 mm port placed in the left anterior axillary line to retract the fat laterally away from the stomach, and a 5 mm port placed midline below the xiphoid process to be used as a liver retractor. The gastric tissue was exposed and was surrounded by adhesions. The adhesions were released using harmonic and hook diathermy to access the GBF site. A fistulojejunostomy was initially intended, but it wasn’t feasible due to inflamed and edematous fistula edges. Consequently, refashioning of the gastric fistula’s edges and invagination using a graham patch was done. Methylene blue test was negative. Then we proceeded with preforming a mini gastric bypass through the usual technique. Gastric pouch was 15 cm in length. After counting of the small bowel from duodenojejunal flexure 180 cm, gastrojejunostomy was done using linear 60 mm purple stapler. Methylene blue was repeated and showed no leak. Postoperatively, the patient was transferred to the ward and recovered smoothly and was discharged home on day 6. A series of gastrographin studies were done with absence of leakage up to 10 months (Fig. 4).

## 3 Discussion

As aforementioned, LSG has become a very popular procedure. Due its simple technique, rare complications might be overseen. Gastric leaks are a fairly uncommon complication with rates that can reach up to 8% [1]. Classification of gastric leaks, as proposed by Csendes A et al. [6], can include any of the following classes: Class I (the subclinical type): Presence of leakage without early septic complications. Class II (the clinical type): Presence of leakage with early septic complications. Another classification, proposed by Antoine Abou Rached et al. [4], was by the site of leakage. Multiple studies were reviewed in their paper, and it was found that most leaks occur at the proximal one third.

Continuation of the leak might lead to the development of a fistula [2]. Developing a fistula is rare, with rates ranging from 2% to 4% [3]. In comparison to other types of fistulas, gastrobronchial fistulas are fairly uncommon. Most of what was found in the literature is that patients presenting with fistulas were mainly treated endoscopically [4,7,8]. When it comes to surgical management, performing a Roux-en-Y fistulojejunostomy was mainly recommended [9,10]. As in our case, patients with gastrobronchial fistula will most likely have underwent LSG as their primary surgery [2].

GBF can have an insidious presentation with non-specific respiratory symptoms which can delay the diagnosis and deteriorate the patient’s condition. That includes chronic cough, shortness of breath, pleuritic chest pain and recurrent pneumonia. Our patient presented with a history of staple line leakage, productive cough and undocumented fever. Recurrent respiratory complaints in a post-bariatric-surgery patient should direct treating doctors toward further investigation. One of the points that can be concluded from Silva et al.’s paper [2], is that most cases of GBF had a history of gastric leak.

Different imaging modalities have been used to verify the diagnosis of GBF including CT, swallow study and endoscopy. X-Ray can be used as an initial imaging toward a definitive choice of imaging. CT scan is suitable for the assessment of lung pathologies and abscesses identification and drainage. Bronchoscopy works by visualizing orally administered methylene blue in the distal bronchi [11]. Yet, a couple of studies showed it to be ineffective [12,13]. Barium study (gastrograffin), on the other hand, shows to be an effective modality in identifying the fistula by demonstrating the barium passage from the gastric pouch into the bronchial tree [14].

Given the rarity of GBF development, a standard way of treating such a complication has not been established. And this necessitated that each case should be tailored independently. Nevertheless, conservative treatment should always be considered, as well as endoscopic management. It has been reported that endoscopic management offered a better option with less complication [2]. Administration of antibiotics is necessary as well, in cases of a coexisting lung infection. And if need be, a CT-guided percutaneous drainage in case of abscess development [11]. As previously mentioned, performing a Roux-en-Y fistulojejunostomy was mainly recommended in the literature.

## 4 Conclusion

With the increasing rates of sleeve gastrectomy, the need for studying such complications and their optimal treatment option is of great importance. Moreover, we recommend researches at different institutions to assess mini-gastric bypass as a modality for the treatment of GBF. Gastrobronchial fistula is a rare yet serious complication of bariatric surgery. Reaching a diagnosis can be difficult due to vague symptoms at the time of presentation. Surgical management commonly showed to be an effective way of managing such patients. Yet, conservative and endoscopic management should be considered initially.

## Funding

None.

## Ethical approval

Ethical approval is not applicable.

## Consent

Written informed consent was obtained from the patient for publication of this case report and accompanying images. A copy of the written consent is available for review by the Editor-in-Chief of this journal on request.

## Author contribution

Dr Alsayyari and Dr Almuhaideb contributed in medical record review, literature search, and writing of the draft. Prof Alnaami and Dr Alharbi contributed towards review of the paper.

## Registration of research studies

None.

## Guarantor

All authors have read and approved the manuscript and accept full responsibility for the work.

## Provenance and peer review

Not commissioned, externally peer-reviewed.

## Declaration of Competing Interest

Authors have no conflict of interest to disclose.
